# The role of the microRNA-146a/complement factor H/interleukin-1β-mediated inflammatory loop circuit in the perpetuate inflammation of chronic temporal lobe epilepsy

**DOI:** 10.1242/dmm.031708

**Published:** 2018-03-01

**Authors:** Tao-Ran Li, Yan-Jie Jia, Chao Ma, Wen-Ying Qiu, Qun Wang, Xiao-Qiu Shao, Rui-Juan Lv

**Affiliations:** 1 Department of Neurology, Beijing Tiantan Hospital, Capital Medical University; China National Clinical Research Center for Neurological Diseases, 6 TianTanXiLi, Dongcheng District, Beijing, 100050, China; 2 Department of Neurology, The First Affiliated Hospital of Zhengzhou University, Zhengzhou University, 1 East Road of JianShe, Erqi District, Zhengzhou, 450052, China; 3 Institute of Basic Medical Sciences, Neuroscience Center, Chinese Academy of Medical Sciences, Department of Human Anatomy, Histology and Embryology, School of Basic Medicine, Peking Union Medical College,1 Shuai Fu Yuan, Dongcheng District, Beijing, 100730, China

**Keywords:** Temporal lobe epilepsy, Inflammation, Interleukin-1β, Complement factor H, MicroRNA-146a

## Abstract

Increasing evidence indicates that neuroinflammation plays a crucial role in the pathogenesis of temporal lobe epilepsy (TLE). However, it is unclear how the perpetuate inflammation develops. Some recent studies have suggested the possible involvement of microRNA-146a (miR-146a) in the modulation of inflammatory signaling occurring in TLE. To understand how miR-146a modulates inflammatory signaling in TLE, we investigated the role of interleukin-1β (IL-1β), miR-146a and human complement factor H (CFH) in the perpetuate inflammation in rat models of chronic TLE and U251 cells. We found that enhancive miR-146a could upregulate the expression of IL-1β and downregulate the expression of CFH, whereas reductive miR-146a could downregulate the expression of IL-1β and upregulate the expression of CFH, in hippocampi of chronic TLE rat models. Meanwhile, enhancive miR-146a could increase the abnormal wave forms in the chronic TLE rat models. Additionally, enhancive IL-1β could feedback downregulate the expression of CFH, upregulate the expression of miR-146a and increase the abnormal wave forms in chronic TLE rat models. After *CFH* gene knockdown in U251 cells, enhancive miR-146a did not upregulate the expression of IL-1β. In summary, this study shows that enhancive miR-146a can upregulate the inflammatory factor IL-1β in chronic TLE by downregulating CFH, and that upregulation of IL-1β plays an important feedback-regulating role in the expression of miR-146a and CFH, forming a miR-146a–CFH–IL-1β loop circuit that initiates a cascade of inflammation and then leads to the perpetuate inflammation in TLE. Therefore, modulation of the miR-146a–CFH–IL-1β loop circuit could be a novel therapeutic target for TLE.

## Abstract (precis)

**Summary:** The microRNA-146a–complement factor H–interleukin-1β loop circuit might initiate a cascade of inflammation, leading to the perpetuate inflammation in temporal lobe epilepsy.

## INTRODUCTION

Epilepsy is a neurological disorder that is characterized by a predisposition to generate seizures associated with neurobiological, psychological, cognitive and linguistic problems (Duncan et al., 2006). There are 65 million people worldwide with epilepsy (with developing countries accounting for 80% of all cases), producing a heavy health and economic burden (Hirtz et al., 2007; Winkler et al., 2007). World Health Organization (WHO) research has shown that epilepsy accounts for 1% of the global disease burden, which is equivalent to the burden of male lung cancer or female breast cancer patients (Engel, 2008). The epileptogenesis might involve ion channels, synaptic remodeling, glial cell proliferation and neuronal death (DeFelipe, 1999; McNamara et al., 2006; Wetherington et al., 2008; Jimenez-Mateos et al., 2012). However, the anti-epilepsy measures targeting these mechanisms still cannot produce satisfactory effects (Pitkänen and Lukasiuk, 2011). It is estimated that one-third of epilepsy patients will develop drug refractory epilepsy, even with the introduction of some new anti-epileptic drugs over the past two decades (Laxer et al., 2014). The failure of drug treatment might be caused by an incomplete understanding of the pathophysiological mechanism underlying drug refractory epilepsy; therefore, it is important to explore this mechanism further.

Temporal lobe epilepsy (TLE) is the most prevalent form of pharmacoresistant focal epilepsy and often accompanies hippocampus sclerosis (HS) (Sendrowski and Sobaniec, 2013; Chatzikonstantinou, 2014). In recent years, evidence from clinical and experimental studies has indicated that brain inflammation is an intrinsic feature of the hyperexcitable pathologic brain tissue in pharmacoresistant epilepsy of different etiologies (Vezzani et al., 2011a). A meta-analysis investigated various inflammatory mediators in human epilepsy of various media and concluded that inflammatory pathways were involved in epilepsy (de Vries et al., 2016). Above all, increased interleukin-1β (IL-1β) expression was found in the brain tissues of TLE patients compared with autopsy controls (Kan et al., 2012a; Fiala et al., 2013), especially in patients with HS (Ravizza et al., 2008; Kan et al., 2012a; Omran et al., 2012). Furthermore, the basal level of IL-1β was barely detectable in the intact rat brain (Plata-Salamán et al., 2000; Lehtimäki et al., 2003), but IL-1β mRNA and protein expression rapidly increased in the cortex, hippocampus and other regions following various experimenter-induced seizures (Minami et al., 1990; Donnelly et al., 1999; Vezzani et al., 1999, 2002; De Simoni et al., 2000; Eriksson et al., 2000; Plata-Salamán et al., 2000; Svensson et al., 2001; Okada et al., 2002; Lehtimäki et al., 2003; Williams et al., 2003; Oprica et al., 2006; Pernot et al., 2011; Omran et al., 2012). Intrahippocampal injection of IL-1β in rodent epilepsy models can deteriorate and prolong both electrographic and behavioral seizure activity (Vezzani et al., 1999, 2002). Perpetuate inflammation plays a key role in the pathophysiology of TLE (Vezzani et al., 2011b), and can reduce the threshold of epileptic seizures (Ravizza et al., 2011), potentiate the excitability of neurons (Galic et al., 2008), lead to neuronal death, affect the physiological function of glial cells and increase the permeability of the blood brain barrier (Vezzani and Granata, 2005), resulting in an increase in the frequency and degree of seizures (Vezzani et al., 1999, 2002; De Simoni et al., 2000; Dubé et al., 2005; Ravizza et al., 2011). Blocking the pro-inflammatory signaling pathway with specific drugs can significantly reduce seizures (Marchi et al., 2011; Maroso et al., 2011a,b; Abraham et al., 2012). This shows that the perpetuate inflammation is not an epiphenomenon of epilepsy, but it can cause and drive the progress of epilepsy. Therefore, exploring the reasons for the perpetuate inflammation might help us to better understand epileptogenesis and develop novel treatment methods.

During the past several years, microRNAs (miRNAs) have emerged as important post-transcriptional regulators of gene expression, providing a completely new level of controlling gene expression. miRNAs are short (20-23 nucleotides), noncoding RNAs that recognize partially complementary target sequences in selected mRNAs, and predominantly inhibit protein expression by either destabilizing their mRNA targets or by inhibiting protein translation (Ambros, 2004; Bartel, 2004; Kosik, 2006; Eulalio et al., 2008). MicroRNA-146a (miR-146a) is a molecule that has been studied by many researchers, and might be involved in epileptogenesis by regulating the inflammatory response. miR-146a has been identified as a key regulator in the feedback system, and its expression was upregulated when induced by nuclear factor kappa B subunits through a myeloid differentiation factor 88-dependent pathway, which in turn could downregulate the levels of interleukin-1 (IL-1) receptor-associated protein kinases-1 and -2 and tumor necrosis factor (TNF) receptor-associated factor 6, downstream of Toll-like and cytokine receptors, reducing the activity of this inflammatory pathway (Taganov et al., 2006; Hou et al., 2009). miR-146a was significantly upregulated in the hippocampi obtained from patients with TLE, as well as in experimental TLE rats (Aronica et al., 2010; Song et al., 2011; Hu et al., 2012; Omran et al., 2012; He et al., 2016), suggesting the possible role of miR-146a in epileptogenesis.

Complement factor H (CFH) is an important and potent inhibitor of the amplification cascade of the alternative pathway of complement activation (Makou et al., 2013). Systemic CFH deficits are conducive to excessive and pathogenic complement activation associated with increased complement activity in healthy host cells, autoimmunity, host tissue damage, and a sustained or chronic inflammatory response (Alexander and Quigg, 2007; Griffiths et al., 2009; Song et al., 2009; Deangelis et al., 2011; Li et al., 2012). In Alzheimer's disease (AD), CFH has been shown to be significantly downregulated (Lukiw et al., 2008; Hill et al., 2009; Pogue et al., 2009; Li et al., 2011, 2012; Lukiw, 2012) and might be a candidate plasma biomarker (Song et al., 2009). In a rat model of TLE, researchers proved that the persistence of complement activation could contribute to a sustained inflammatory response and destabilize the neuronal networks involved (Aronica et al., 2007).

Bioinformatics analysis indicated that miR-146a had overlapping target recognition sites within the CFH mRNA 3′-untranslated region (3′-UTR; 5′-TTTAGTATTAA-3′) (Lukiw et al., 2012b; He et al., 2016). Further studies showed that miR-146a can downregulate the expression of CFH (Lukiw et al., 2008; Hill et al., 2009; Pogue et al., 2009; Li et al., 2011; Lukiw, 2012; Lukiw et al., 2012a; He et al., 2016), and these effects were suppressed by incubation with an antisense oligonucleotide to miR-146a (Lukiw et al., 2008, 2012a). miR-146a-mediated CFH deficits are conducive to excessive complement pathway activation associated with autoimmunity and a sustained inflammatory response. However, almost all references were developed in an AD-related field (Lukiw et al., 2008; Hill et al., 2009; Pogue et al., 2009; Li et al., 2011; Lukiw, 2012; Lukiw et al., 2012a,b). As inflammation and epilepsy are closely related, the decreased expression of CFH appears to contribute to dysregulated complement signaling with significant pro-inflammatory consequences such as epileptogenesis (He et al., 2016).

The dysregulated miRNAs in TLE patients prominently target inflammatory mediators (Kan et al., 2012b). Several studies have verified that IL-1β represents a major pro-inflammatory cytokine that can promote the expression of miR-146a in cell experiments (Taganov et al., 2006; Nakasa et al., 2008; Iyer et al., 2012). In particular, Iyer et al. showed a significant upregulation of miR-146a in the astrocytoma cell line or cultured human astrocytes when exposed to IL-1β stimulation (Iyer et al., 2012); thus it is possible that expression of miR-146a in astrocytes could function in fine-tuning the inflammatory response triggered by IL-1β (Aronica et al., 2010). Omran et al. also speculated that the IL-1β–miR-146a loop circuit could be a new target for antiepileptic therapy, examining the dynamic expression of IL-1β and miR-146a in TLE rat models (Omran et al., 2012).

Based on the above, we hypothesized that enhancive miR-146a can lead to an increase in IL-1β by downregulating CFH expression, and increased IL-1β can upregulate miR-146a expression further, forming the miR-146a–CFH–IL-1β loop circuit that initiates a cascade of inflammation and leads to the perpetuate inflammation in TLE. In the present study, we investigated the expression and regulation of miR-146a, CFH and IL-1β, as well as the role of the miR-146a–CFH–IL-1β loop circuit, in perpetuate inflammation in a chronic TLE rat model.

## RESULTS

### Status epilepticus induced by intrahippocampal kainic acid

**Fig. 1.**
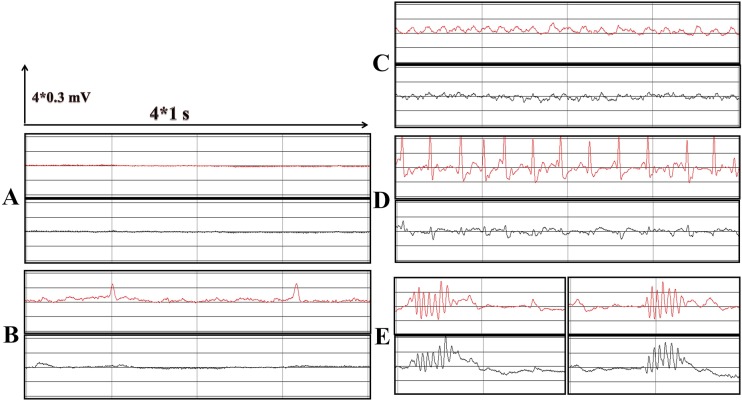
**EEG recordings before and after KA administration.** The distance between two longitudinal lines represents 1 s and the distance between two horizontal lines represents 0.3 mV. Red represents the left hemisphere, and black represents the right hemisphere. (A) Baseline EEG before KA injection; waves with low amplitude and high frequency can be seen. (B) Sporadic sharp waves or spike waves can be seen at the inter-ictal phase. (C,D) A generalized burst of sharp or spike waves appeared after a period of slow-wave rhythms, which accompanied the motor symptoms. (E) Successive spike waves appeared with wet-dog shakes.

After microinjecting kainic acid (KA) into the CA3 region of the posterior hippocampus, the behavioral seizures of the rat models were continuously monitored for 24 h with video recording. The videos were confirmed by three experienced neurosurgical researchers. Generally, within 2 h after waking from anesthesia, rats were characterized by continuous limbic seizure activity (stereotyped sniffing, head nodding, gnawing, behavioral arrest, wet-dog shakes, straub tail, decreased responsiveness), which was repeatedly interrupted by secondarily generalized convulsive seizures. This status lasted more than 40 min in all rats, and the rats showed a state of exhaustion after status epilepticus (SE). The electrode-implanted rats also received electroencephalogram (EEG) monitoring during the first 24 h. We saw various abnormal waves in the acute phase of TLE. The typical and frequent abnormal waves are shown in Fig. 1B and E, respectively. During generalized motor seizures, we can see continuous slow waves (Fig. 1C), followed by a burst of spike wave rhythms (Fig. 1D).

At week 5 and 6 (chronic phase) post-SE, the rats were video monitored and observed again. The electrode-implanted rats also received EEG monitoring. The EEG displayed normal wave forms (Fig. 1A) in most cases. However, sometimes we observed continuous slow waves (Fig. 1C), followed by a burst of spike wave rhythms (Fig. 1D) with limbs clonus. Wet-dog shakes were also seen (Fig. 1E). Rats with seizures above Racine Class III and nonconvulsive seizures that had signs of seizure on the EEG also received further study.

### Decreased levels of CFH were detected in the hippocampi of TLE patients

**Fig. 2.**
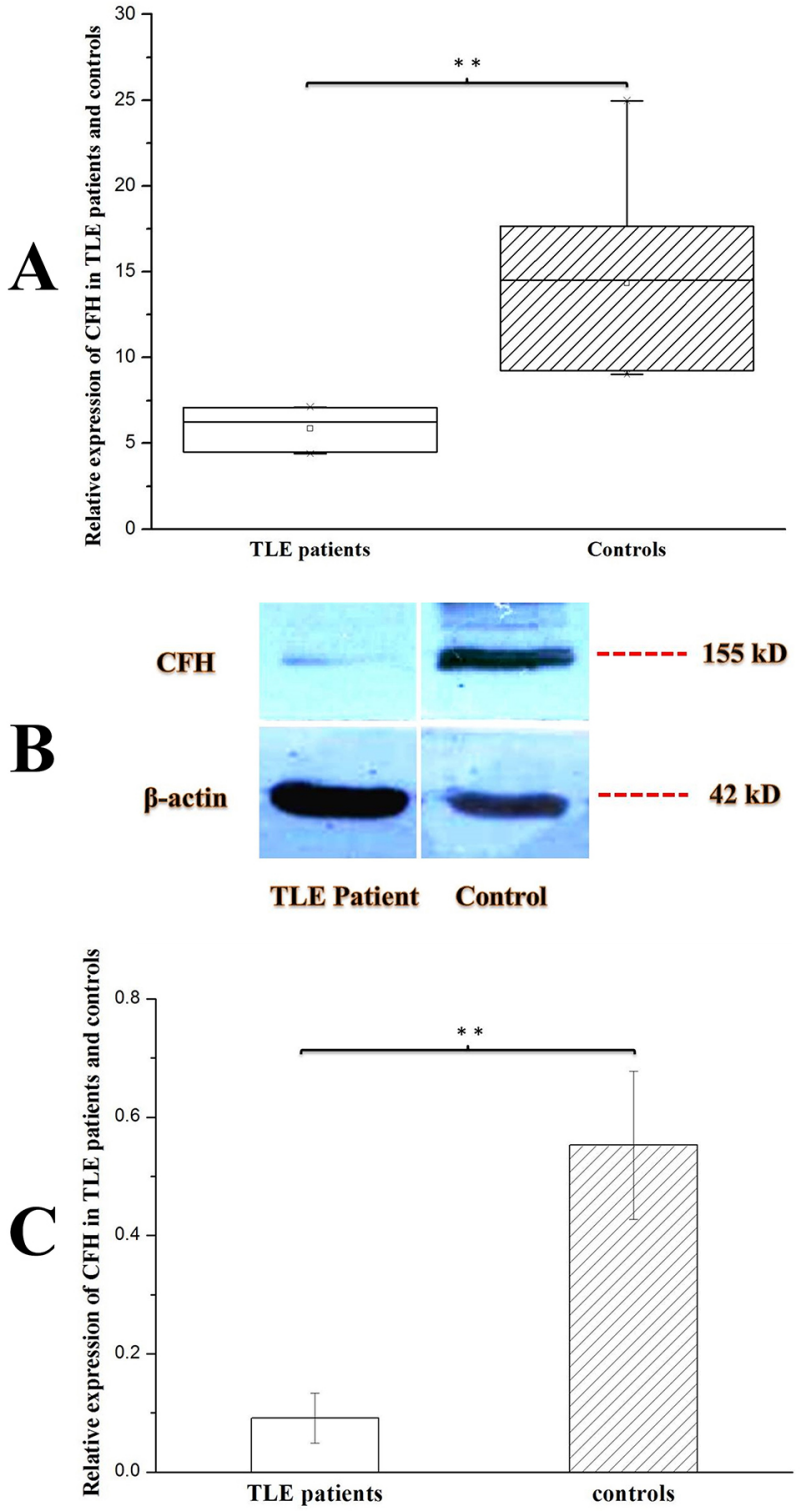
**Expression of CFH in the hippocampi of TLE patients (*n*=7) and controls (*n*=8).** Statistical analysis was conducted by one-way ANOVA. **P<0.01. Values represent means±s.d. WB quantitation was performed using ImageJ software. (A) *CFH* expression in the human hippocampus was detected by qRT-PCR. The expression was normalized to *β-actin* in each tissue. *CFH* expression was obviously reduced in TLE patients compared with controls. (B) CFH and β-actin protein expression in the hippocampus of a TLE patient compared with a control patient confirmed the qRT-PCR results. (C) Relative grayscales (CFH compared with β-actin) of TLE (*n*=3) and control (*n*=3) patients showed similar results to those from qRT-PCR.

A previous study showed that miR-146a can suppress CFH expression to contribute to the inflammatory pathology of AD (Lukiw et al., 2008). Therefore, we were interested in determining CFH expression levels in the hippocampi of TLE patients. We observed that hippocampal expression of CFH was markedly decreased in the TLE patients compared with the control patients (*P*<0.01) (Fig. 2).

### Effects of miR-146a agomir/antagomir on IL-1β and CFH hippocampal expression in chronic TLE rat models

In the chronic TLE rat models, rats were injected with miR-146a agomir, miR-146a antagomir or miR-146a control in the hippocampal region at week 7 (chronic phase) post-SE. The miR-146a control is originated from *Caenorhabditis elegans* and has no homology with the rat genome, which is the contrast of miR-146a agomir or miR-146a antagomir. Hippocampi were collected 48 h after injection. miR-146a was detected using real-time quantitative PCR (qRT-PCR) analysis. We found that miR-146a expression was upregulated in the agomir group compared with the antagomir or control group (*P*<0.01). However, miR-146a expression was suppressed in the antagomir group compared with the control group (*P*<0.01) (Fig. S1). These results showed that miR-146a agomir/antagomir can upregulate/downregulate miR-146a expression, respectively.

**Fig. 3.**
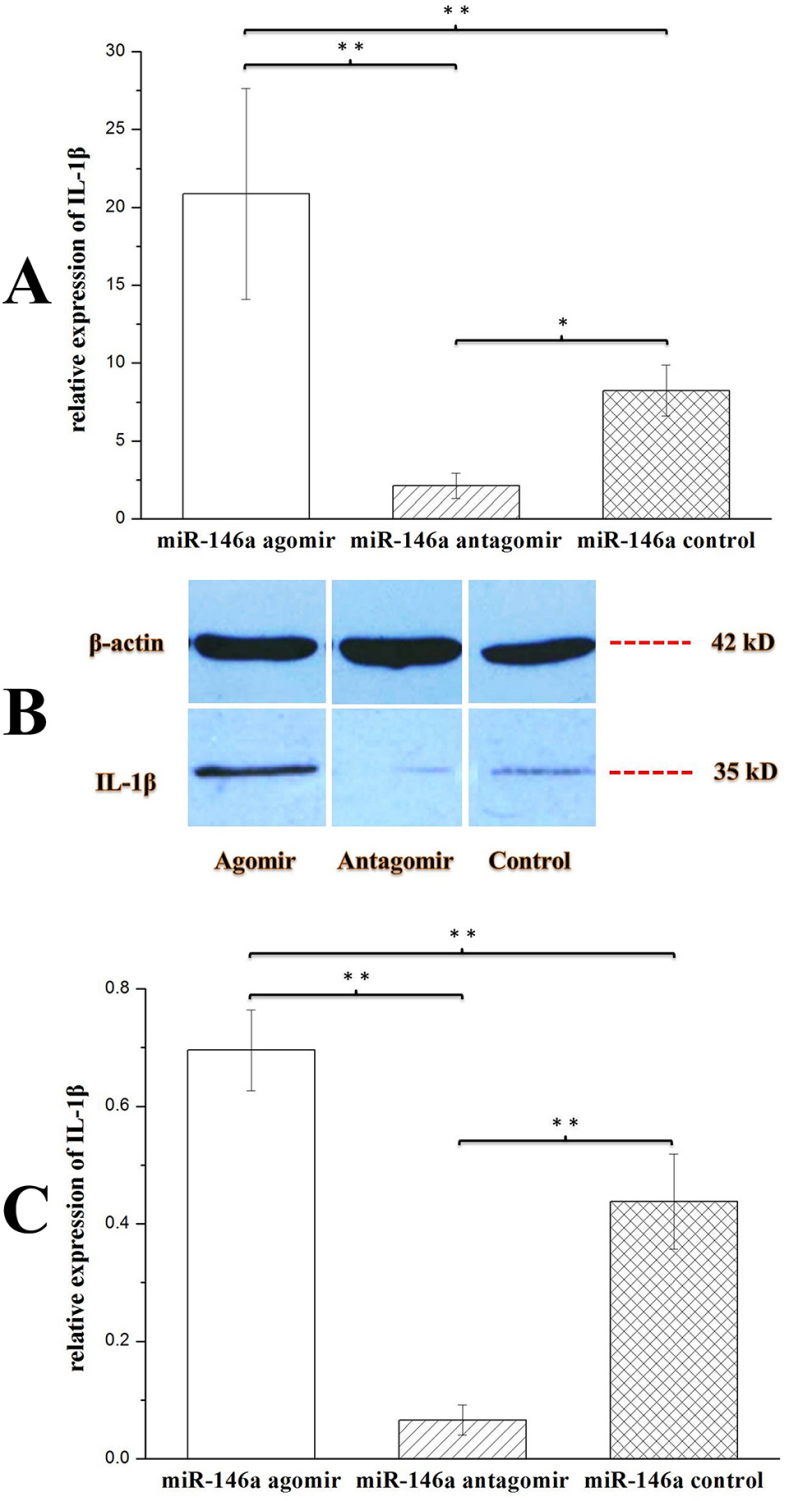
**Effects of miR-146a agomir/antagomir on IL-1β hippocampal expression in chronic TLE rat models.** Expression of IL-1β in the hippocampi of chronic TLE rats after injection with miR-146a agomir (*n*=10), miR-146a antagomir (*n*=10) or control (*n*=10). Statistical analysis was conducted by one-way ANOVA followed by LSD test. *P<0.05; **P<0.01. Values represent means±s.d. WB quantitation was performed using ImageJ software. At week 5 and 6 (chronic phase) post-SE, all rats were video monitored, and electrode-implanted rats also received EEG monitoring. All rats experienced seizures, and abnormal seizure waves were found in electrode-implanted rats. Rats were injected with miR-146a agomir, miR-146a antagomir or control at week 7 (chronic phase) post-SE, and hippocampal tissues were collected 48 h later. (A) Expression of *IL-1β* was detected by qRT-PCR. The expression was normalized to *β-actin* in each tissue. *IL-1β* expression was upregulated in the miR-146a agomir group compared with the miR-146a antagomir or control group, and downregulated in the miR-146a antagomir group compared with the miR-146a control group. (B) IL-1β and β-actin protein expression in the hippocampi of chronic TLE rats after injection with miR-146a agomir, miR-146a antagomir or control confirmed the qRT-PCR results. (C) Relative grayscales (IL-1β compared with β-actin) of agomir group rats (*n*=3), antagomir group rats (*n*=3) and control group rats (*n*=3) showed similar results to those from qRT-PCR.

To understand the effects of miR-146a on inflammation in chronic TLE rat models, the rats were injected with miR-146a agomir or miR-146a antagomir at week 7 (chronic phase) post-SE. Hippocampi were collected 48 h after injection. IL-1β was detected using qRT-PCR and western blotting (WB). As shown in Fig. 3A, *IL-1β* expression was upregulated in the miR-146a agomir group compared with the control group or miR-146a antagomir group (*P*<0.01), and was lower in the miR-146a antagomir group than in the control group (*P*<0.05). These results were confirmed by WB (Fig. 3B,C). Taken together, the results showed that miR-146a had an important effect on inflammation in the hippocampi of chronic TLE rats.

**Fig. 4.**
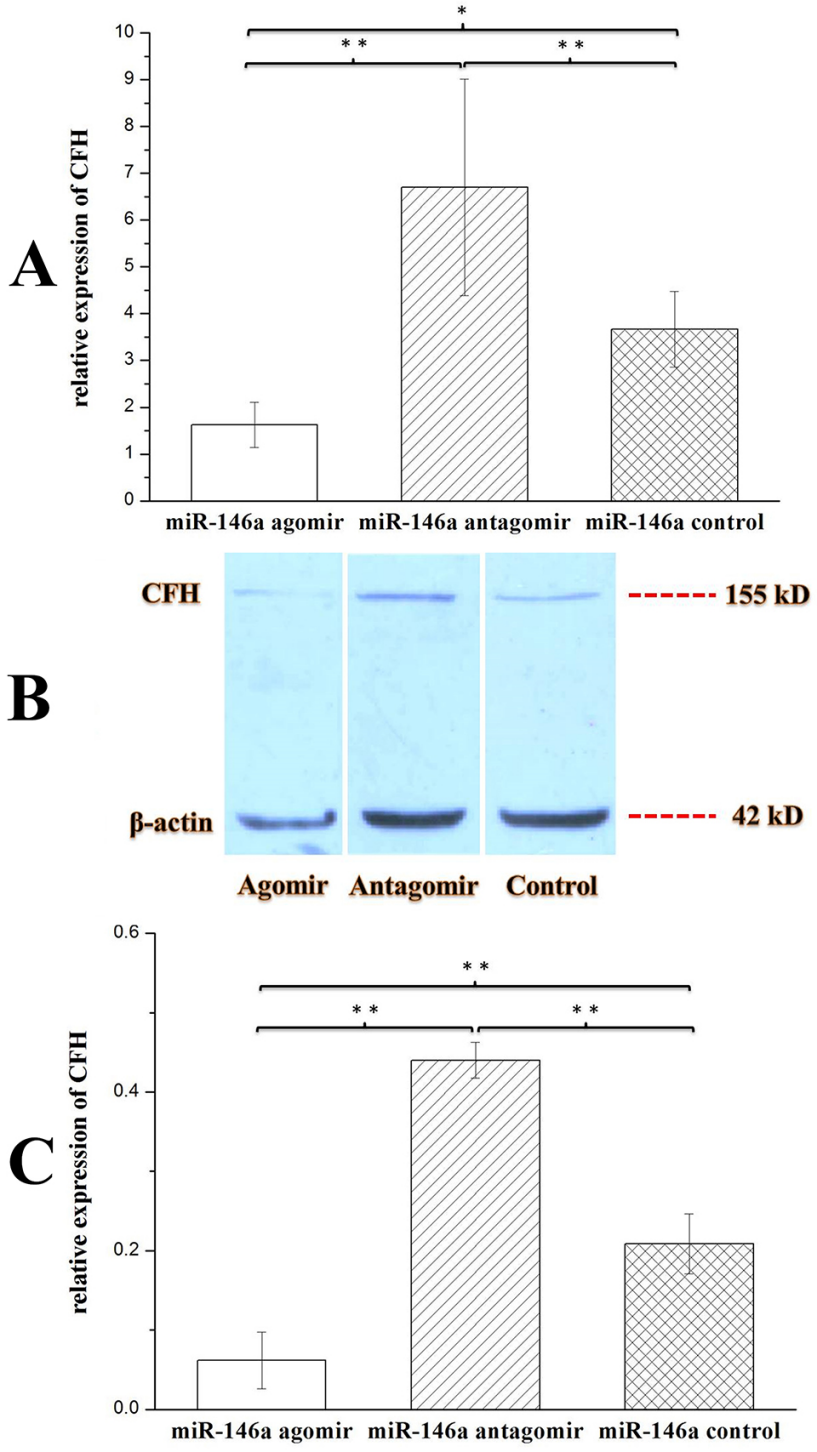
**Effects of miR-146a agomir/antagomir on CFH hippocampal expression in chronic TLE rat models.** Expression of CFH in the hippocampi of chronic TLE rats after injection with miR-146a agomir (*n*=10), miR-146a antagomir (*n*=10) or control (*n*=10). Statistical analysis was conducted by one-way ANOVA, followed by LSD test. *P<0.05; **P<0.01. Values represent means±s.d. WB quantitation was performed using ImageJ software. At week 5 and 6 (chronic phase) post-SE, all rats were video monitored, and electrode-implanted rats also received EEG monitoring. All rats experienced seizures, and abnormal seizure waves were found in electrode-implanted rats. Rats were injected with miR-146a agomir, miR-146a antagomir or control at week 7 (chronic phase) post-SE, and hippocampal tissues were collected 48 h later. (A) Expression of *CFH* was detected by qRT-PCR. The expression was normalized to *β-actin* in each tissue. *CFH* expression was downregulated in the miR-146a agomir group compared with the miR-146a antagomir or control group, and upregulated in the miR-146a antagomir group compared with the control group. (B) CFH and β-actin protein expression in the hippocampi of chronic TLE rats after injection with miR-146a agomir, miR-146a antagomir or control confirmed the qRT-PCR results. (C) Relative grayscales (CFH compared with β-actin) of agomir group rats (*n*=3), antagomir group rats (*n*=3) and control group rats (*n*=3) showed similar results to those from qRT-PCR.

To determine the ability of miR-146a to regulate CFH expression in chronic TLE rat models, the rats were injected with miR-146a agomir or miR-146a antagomir at week 7 (chronic phase) post-SE. Hippocampi were collected 48 h after injection. CFH was detected using qRT-PCR and WB. As shown in Fig. 4A, *CFH* expression was lower in the miR-146a agomir group than in the control group (*P*<0.05) or miR-146a antagomir group (*P*<0.01). *CFH* expression was higher in the miR-146a antagomir group than in the control group (*P*<0.01). These results were confirmed by WB (Fig. 4B,C), and indicated that miR-146a can regulate CFH expression levels in the hippocampi of chronic TLE rats. Considering these and the results presented in Figs 2 and 3, we speculated that CFH might be involved in the inflammatory pathology of chronic TLE.

### miR-146a increased the abnormal wave forms of chronic TLE rats

**Fig. 5.**
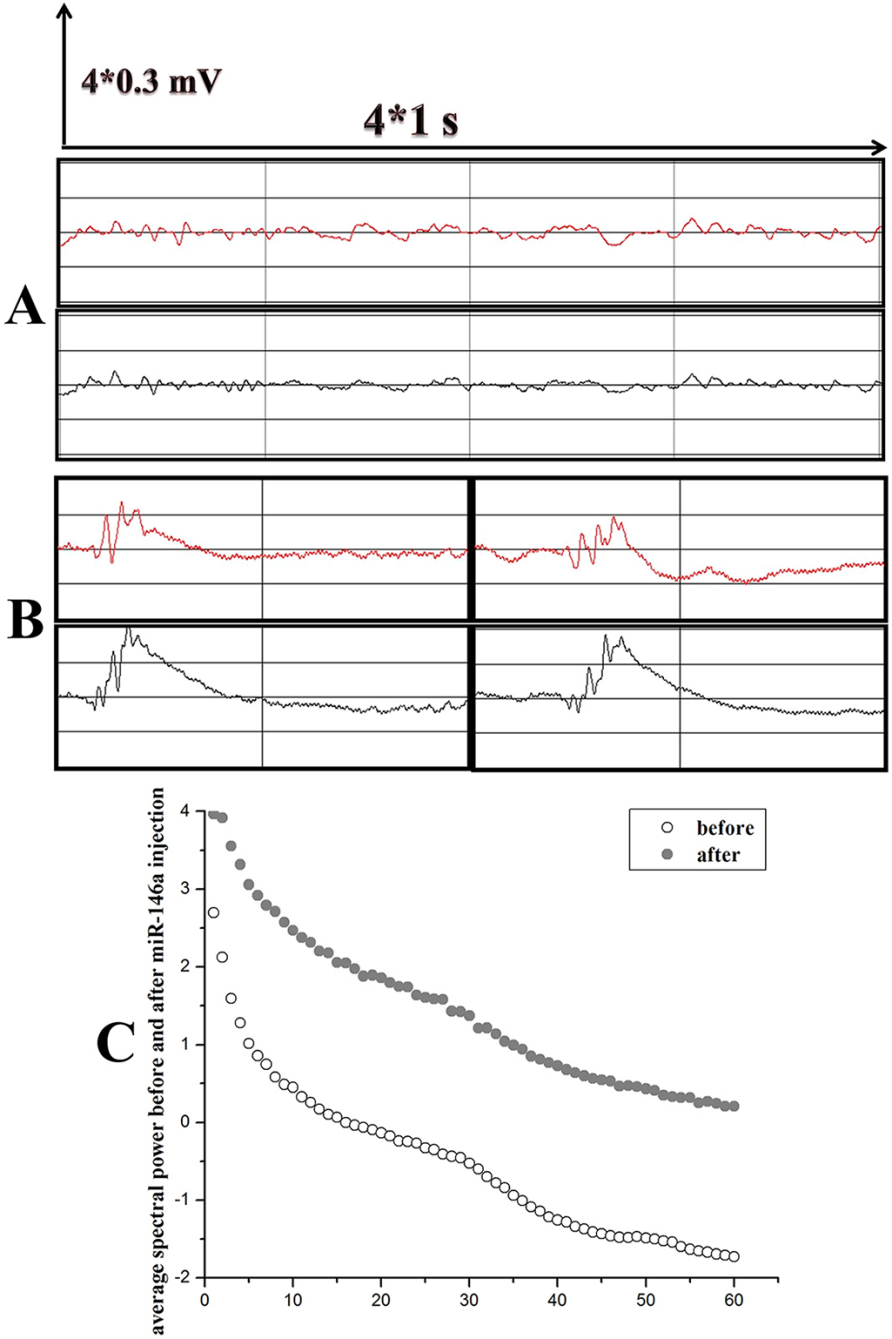
**EEG recordings of chronic TLE rats after miR-146a agomir administration.** The distance between two longitudinal lines represents 1 s and the distance between two horizontal lines represents 0.3 mV. Red represents the left hemisphere and black represents the right hemisphere. (A) In the chronic phase of TLE, sharp waves can be seen frequently. (B) Successive spike waves appeared with wet-dog shakes. (C) EEG performance was monitored at the same time in the afternoon, and 30-min samples of electrical activity were selected for spectral power analysis at the four time points (1 day and 2 days before injection, 1 day and 2 days after injection). The open circles represent the average spectral power of all electrode-implanted miR-146a group rats (*n*=3) before injection with miR-146a agomir, and the gray circles indicate the average spectral power after injection. The analysis used logarithmic form expression, and the frequency bands spanned 1-60 Hz. We observed a marked increase in the average spectral power following injection of miR-146a agomir.

To confirm the important role of miR-146a in seizures in chronic TLE rats, we injected rats with miR-146a agomir at week 7 (chronic phase) post-SE. After injection, the behavioral seizures of the rats were continuously monitored for 48 h with video recording. The electrode-implanted rats also received EEG monitoring at the same time before injection. We did not find an obvious increase in convulsive motor seizures following miR-146a agomir injection, but we did observe an obvious increase in the abnormal wave forms (Fig. 5A,B). The average spectral power increased after the miR-146a agomir injection (Fig. 5C).

### IL-1β upregulated miR-146a in the hippocampi of chronic TLE rats

To determine the ability of inflammation to regulate the expression of miR-146a in the hippocampi of chronic TLE rats, the rats were injected with IL-1β at week 7 (chronic phase) post-SE. Hippocampi were collected 48 h after injection. miR-146a was detected using qRT-PCR. As shown in Fig. S2, miR-146a expression was upregulated in the IL-1β group compared with the negative control group (*P*<0.01), indicating that increased inflammation could upregulate miR-146a levels in the hippocampi of chronic TLE rats.

### IL-1β downregulated CFH in the hippocampi of chronic TLE rats

**Fig. 6.**
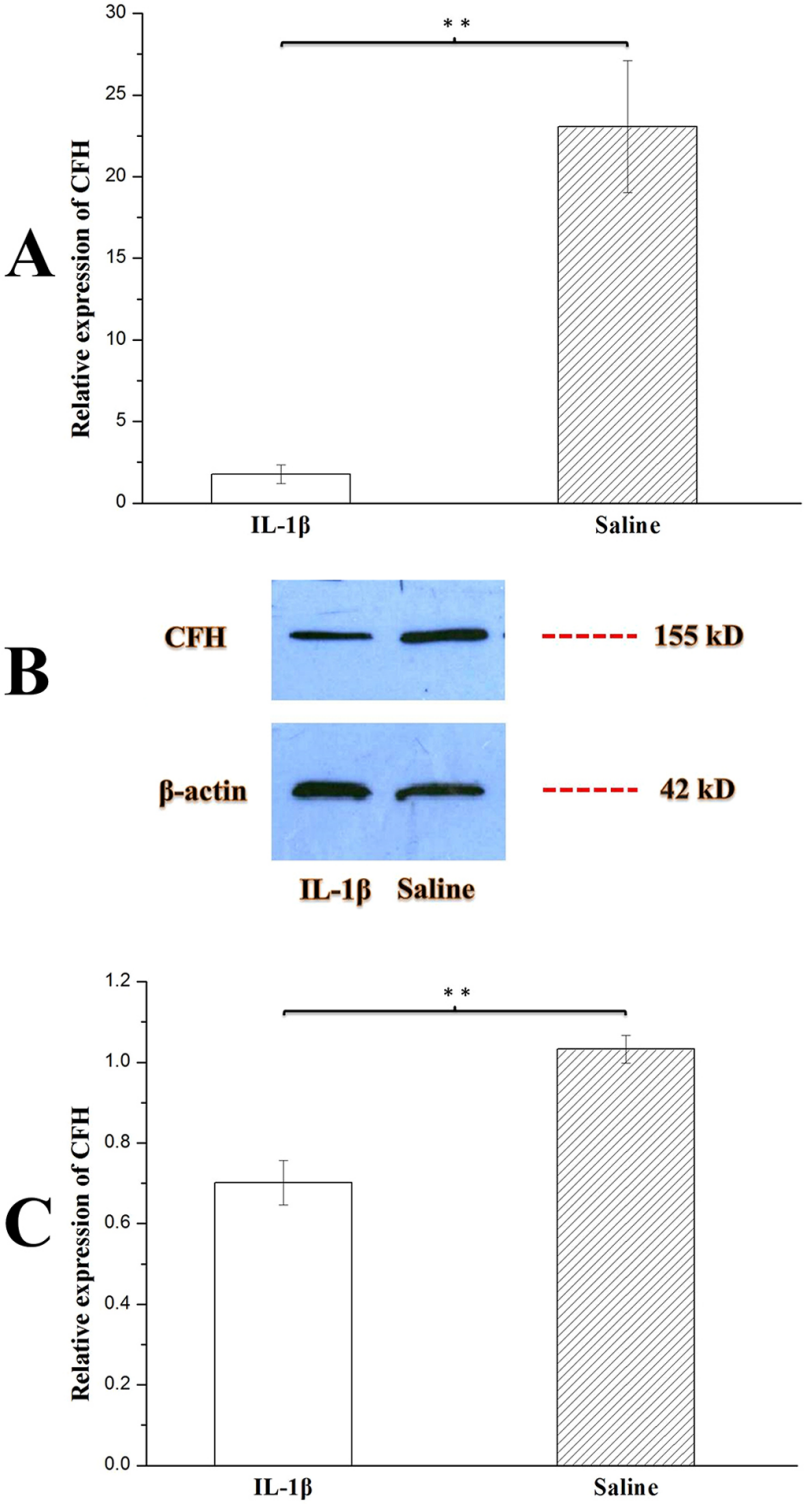
**Expression of CFH in the hippocampi of chronic TLE rats after injection with saline (*n*=10) or IL-1β (*n*=10).** Statistical analysis was conducted by one-way ANOVA. **P<0.01. Values represent means±s.d. WB quantitation was performed using ImageJ software. At week 5 and 6 (chronic phase) post-SE, all rats were video monitored, and electrode-implanted rats also received EEG monitoring. All rats experienced seizures, and abnormal seizure waves were found in electrode-implanted rats. Rats were injected with IL-1β or saline at week 7 (chronic phase) post-SE, and hippocampal tissues were collected 48 h later. (A) *CFH* expression in the hippocampi of chronic TLE rats was detected by qRT-PCR. The expression was normalized to *β-actin* in each tissue. *CFH* expression was downregulated in the IL-1β group compared with the control group. (B) CFH and β-actin protein expression in the hippocampi of chronic TLE rats after injection with IL-1β or saline confirmed the qRT-PCR results. (C) Relative grayscales (CFH compared with β-actin) of IL-1β group rats (*n*=3) and saline group rats (*n*=3) showed similar results to those from qRT-PCR.

To confirm the ability of IL-1β to feedback regulate the expression of CFH in the hippocampi of chronic TLE rats, the rats were injected with IL-1β at week 7 (chronic phase) post-SE. Hippocampi were collected 48 h after injection, and CFH was detected using qRT-PCR and WB. As shown in Fig. 6A, *CFH* expression was lower in the IL-1β group than in the control group (*P*<0.01), which was confirmed by WB (Fig. 6B,C). This result showed that inflammatory factor IL-1β might play an important feedback-regulating role in the expression of CFH in the hippocampi of chronic TLE rats.

### IL-1β increased the abnormal wave forms in chronic TLE rats

**Fig. 7.**
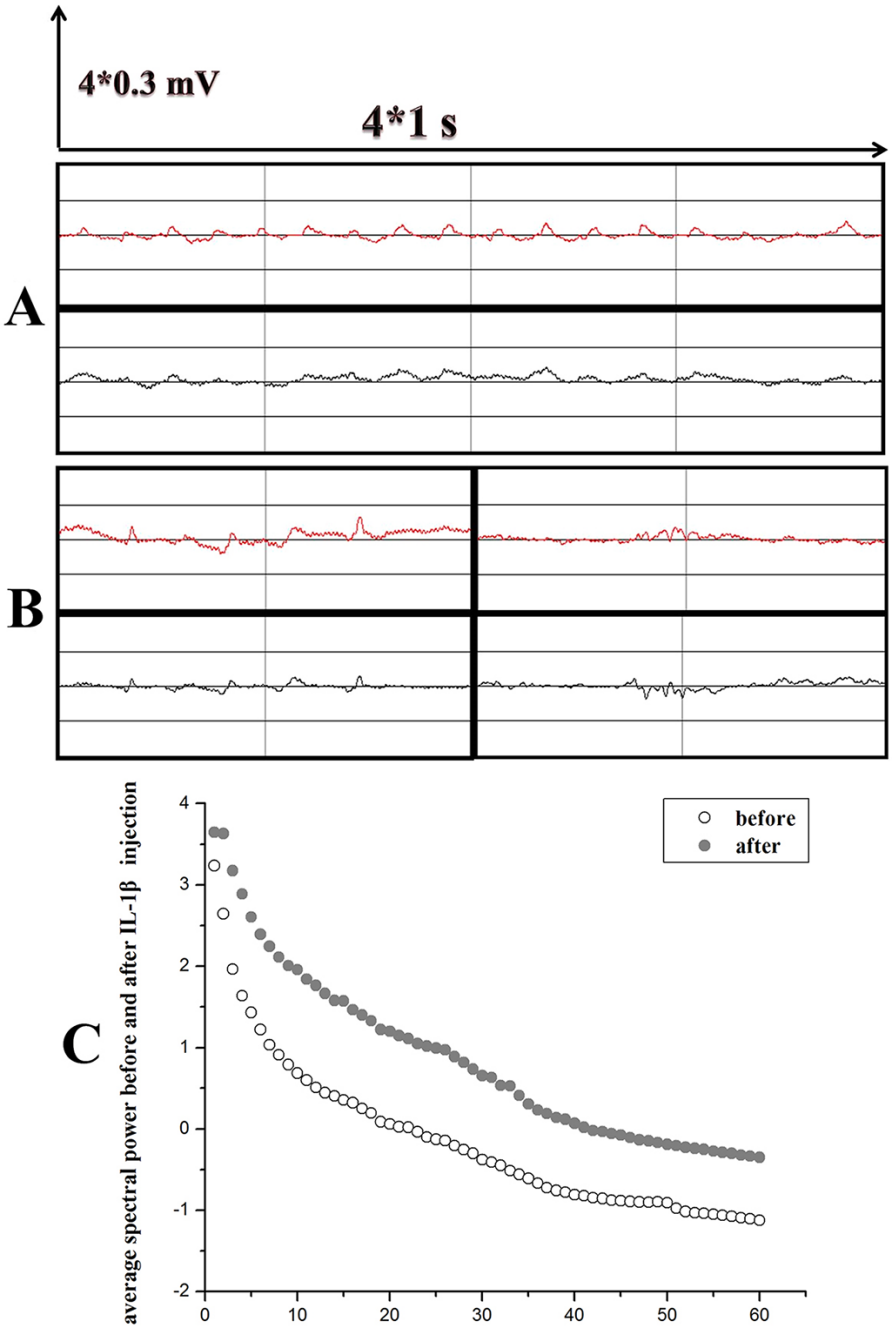
**EEG recordings of chronic TLE rats after IL-1β administration.** The distance between two longitudinal lines represents 1 s and the distance between two horizontal lines represents 0.3 mV. Red represents the left hemisphere and black represents the right hemisphere. (A) In the chronic phase of TLE, the abnormal slow waves can be seen frequently. (B) Sharp waves and poly spike waves can also be seen frequently. (C) EEG performance was monitored at the same time in the afternoon, and 30 min samples of electrical activity were selected for spectral power analysis at the four time points (1 day and 2 days before injection, 1 day and 2 days after injection). The open circles represent the average spectral power of all electrode-implanted IL-1β group rats (*n*=3) before injection with IL-1β, and the gray circles indicate the average spectral power after injection. The analysis used logarithmic form expression, and the frequency bands spanned 1-60 Hz. We observed a marked increase in the average spectral power following injection of miR-146a agomir.

To confirm whether increased inflammation has an effect on seizures in chronic TLE rats, the rats were injected with IL-1β at week 7 (chronic phase) post-SE. After injection, the behavioral seizures of rats were continuously monitored for 48 h with video recording. The electrode-implanted rats also received EEG monitoring at the same time before injection. Interestingly, IL-1β had a similar effect to miR-146a agomir on seizures in the chronic TLE rats. We did not find an obvious increase in convulsive motor seizures following the IL-1β injection, but an obvious increase in the abnormal wave forms was detected by EEG monitoring (Fig. 7A,B). The average spectral power increased after injection with IL-1β (Fig. 7C).

### Enhancive miR-146a could not upregulate expression of IL-1β after *CFH* gene knockdown

**Fig. 8.**
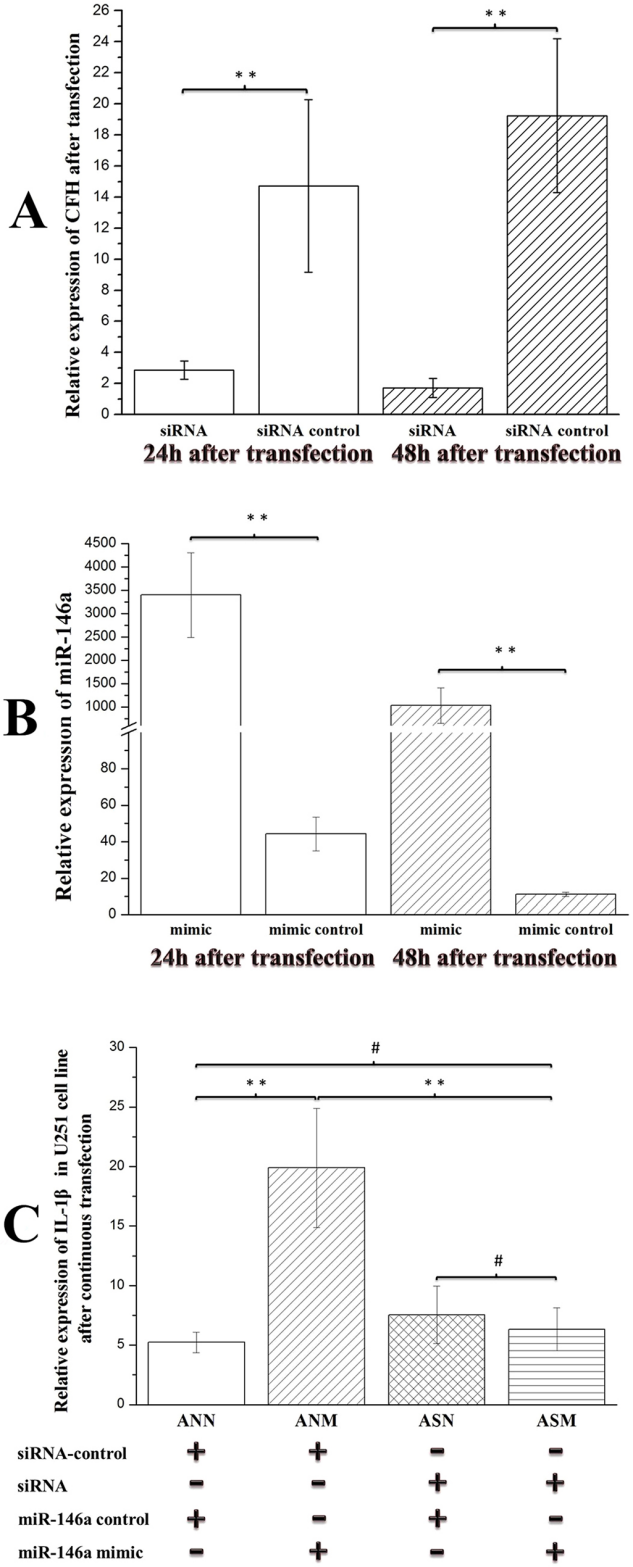
**Expression of IL-1β induced by miR-146a mimic in U251 cells after *CFH* gene knockdown.** Statistical analysis was conducted by one-way (A,B) or factorial design (C) ANOVA. **P<0.01; ^#^*P*>0.05. Values represent means±s.d. (A) Expression of *CFH* in U251 cells after transfection with *CFH*-specific siRNA. The expression was normalized to *β-actin* in each well. Cells were harvested 24 h or 48 h after transfection with *CFH*-specific siRNA, and expression of *CFH* was detected by qRT-PCR. *CFH* expression was markedly reduced in the *CFH*-specific siRNA group compared with the control group. (B) Expression of miR-146a in U251 cells after transfection with miR-146a mimic. The expression was normalized to *U6* in each well. Cells were harvested 24 h or 48 h after transfection with miR-146a mimic, and expression of miR-146a was detected by qRT-PCR. miR-146a expression was markedly increased in the miR-146a mimic group compared with the control group. (C) Expression of *IL-1β* induced by miR-146a mimic in U251 cells after *CFH* gene knockdown. The expression was normalized to *β-actin* in each well. Cells were transfected with miR-146a mimic 24 h after transfection with *CFH*-specific siRNA. The cells were harvested 36 h after transfection and the expression of *IL-1β* was detected by qRT-PCR. *IL-1β* expression was increased in the ANM group compared with the ASM group or ANN group. There were no significant differences in *IL-1β* expression between the ASM group and ANN group, and between the ASM group and ASN group. Details on the specific groups are as follows: ANN (*n*=6, U251 cells transfected with siRNA control and mimic control), ANM (*n*=6, U251 cells transfected with siRNA control and miR-146a mimic), ASN (*n*=6, U251 cells transfected with *CFH*-specific siRNA and mimic control) and ASM (*n*=6, U251 cells transfected with *CFH*-specific siRNA and miR-146a mimic).

As shown in Fig. 8A, *CFH* expression was successfully knocked down by *CFH*-specific siRNA in U251 cells 24 h or 48 h after transfection. Furthermore, transfection with miR-146a mimic increased the expression of miR-146a in U251 cells (Fig. 8B). To explore whether enhancive miR-146a could upregulate the expression of IL-1β by downregulating CFH, U251 cells were transfected with miR-146a mimic after *CFH* gene knockdown. Thirty-six hours after transfection with the mimic, *IL-1β* was detected using qRT-PCR. We found that the miR-146a mimic could increase the expression of *IL-1β*. However, it was interesting that enhancive miR-146a did not upregulate the expression of *IL-1β* in U251 cells after *CFH* gene knockdown (*P*<0.01) (Fig. 8C), which suggested that enhancive miR-146a might upregulate inflammatory factor levels through the CFH pathway.

## DISCUSSION

Extensive research has shown that perpetuate inflammation is an intrinsic feature of TLE, and can cause and drive the progress of epilepsy, resulting in pharmacoresistance (Marchi et al., 2011; Maroso et al., 2011b; Ravizza et al., 2011; Vezzani et al., 2011b). Brain-enriched miR-146a, as a post-transcriptional inflammation-associated miRNA, has been strongly implicated in the regulation of epileptogenesis (Aronica et al., 2010; Omran et al., 2012; He et al., 2016). However, the mechanism of miR-146a regulating the epileptogenesis and progression of TLE is still unclear. Several studies found that miR-146a was associated with sustained inflammation by suppressing CFH in AD (Lukiw et al., 2008, 2012a; Hill et al., 2009; Pogue et al., 2009; Li et al., 2011; Lukiw, 2012), but it is not yet clear whether miR-146a can regulate inflammation via CFH in chronic TLE rats. Therefore, we investigated whether miR-146a can regulate inflammation by the CFH pathway, and whether IL-1β can feedback regulate miR-146a and CFH, forming an inflammation loop circuit that leads to the perpetuate inflammation in chronic TLE rats. Our results show that enhancive miR-146a can upregulate the expression of IL-1β by downregulating CFH. Meanwhile, the enhancive inflammatory factor IL-1β plays an important feedback role in upregulating the expression of miR-146a and downregulating the expression of CFH. Hence, we believe that the miR-146a–CFH–IL-1β loop circuit can lead to the perpetuate inflammation in TLE.

Previous studies showed that miR-146a was significantly upregulated in the hippocampi obtained from patients with TLE, as well as in experimental TLE rats (Aronica et al., 2010; Song et al., 2011; Hu et al., 2012; Omran et al., 2012; He et al., 2016). However, whether enhancive miR-146a can promote inflammation in chronic TLE rats is still unclear. We first initiated the chronic TLE rat models. The eligible rats were verified by convulsive seizures and a burst of spike waves in EEG, or nonconvulsive seizures with EEG seizure waves. After we confirmed that the eligible rats entered the chronic phase by behavioral seizure observation and EEG, the rats were injected with a miR-146a agomir or miR-146a antagomir. We found that enhancive miR-146a could upregulate the expression of IL-1β, whereas reductive miR-146a could downregulate the expression of IL-1β in the hippocampus, suggesting that miR-146a can regulate inflammation in chronic TLE. Recently, one study group demonstrated that downregulating miR-146a can decrease seizure susceptibility in the acute phase in TLE rats (He et al., 2016). Therefore, we wanted to know whether miR-146a has an effect on behavioral seizures and EEG recordings in the chronic phase in TLE rats. We found an obvious increase in the abnormal wave forms after injection with a miR-146a agomir, and the average spectral power increased markedly, though we did not find an obvious increase in convulsive motor seizures compared with before injection, which indicated the enhancement of excitable brain activity.

Furthermore, we determined the effect of miR-146a on CFH and found that enhancive miR-146a could downregulate the expression of CFH, similar to the findings in the AD field (Lukiw et al., 2008, 2012a; Hill et al., 2009; Pogue et al., 2009; Li et al., 2011; Lukiw, 2012). One recent study found that CFH expression was significantly decreased at week 1 and 4 in TLE rats, and that downregulating miR-146a could increase CFH protein levels in the acute phase in TLE rats (He et al., 2016). Additionally, we found that CFH expression was significantly decreased in the hippocampi of TLE patients. Hence, we speculated that miR-146a regulated inflammatory factor IL-1β expression by the CFH pathway in chronic TLE rats. To verify this speculation, we used the U251 cell line to mimic the physiological changes in TLE. We found that enhancive miR-146a could increase the expression of IL-1β in U251 cells before *CFH* gene knockdown. However, enhancive miR-146a could not upregulate the expression of IL-1β in U251 cells after *CFH* gene knockdown, which suggested that enhancive miR-146a might upregulate inflammatory factor levels through the CFH pathway. Recent studies have implicated TLE as a glial disorder rather than a neuronal disorder. In support of this view, several membrane channels, receptors and transporters in astroglial membranes have been found to be deeply altered in the epileptic brain, and these changes alter homeostatic network functions that temporally precede the alterations in neurons (de Lanerolle et al., 2010; Steinhäuser and Boison, 2012; Coulter and Steinhäuser, 2015; Steinhäuser et al., 2016). Astrocytes are also a major source of IL-1β, miR-146a and CFH (Vezzani et al., 2008; Song et al., 2009; Aronica et al., 2010; Li et al., 2011). Therefore, we thought that using the U251 cell line to mimic the physiological changes of TLE was feasible.

Perpetuate inflammation is an important cause of pharmacoresistance in TLE (Marchi et al., 2011; Maroso et al., 2011b; Ravizza et al., 2011; Vezzani et al., 2011b). Previous cell experiments verified that increased IL-1β could upregulate miR-146a expression levels (Taganov et al., 2006; Nakasa et al., 2008; Iyer et al., 2012). Therefore, we wondered whether IL-1β could feedback regulate miR-146a and CFH in chronic TLE rats and then lead to the perpetuate inflammation. After we confirmed that the eligible rats entered the chronic phase by behavioral seizure observation and EEG, the rats were injected with IL-1β. The results revealed that an upregulation of miR-146a expression levels was accompanied by downregulation of CFH. Moreover, the abnormal waves could be seen frequently and the average spectral power increased, similar to the effects caused by enhancive miR-146a in chronic TLE rats, which also indicated the enhancement of brain excitability. These results showed that enhancive inflammatory factor IL-1β might play an important feedback-regulating role in upregulating expression of miR-146a and downregulating the expression of CFH in chronic TLE rats, in turn leading to the perpetuate inflammation in TLE.

This study focused on investigating the role of the miR-146a–CFH–IL-1β loop circuit in regulating inflammation and epileptogenesis in chronic TLE. It was carried out in the chronic phase in TLE rats, which can reflect the long-term course of TLE patients. At week 5 and 6 (chronic phase) post-SE, all rats received video monitoring, and electrode-implanted rats also received EEG monitoring. All the eligible rats experienced seizures, and abnormal waves were found in electrode-implanted rats, which were verified by three experienced neurosurgical researchers. Therefore, the results were real and reliable. In addition, for the first time, we detected that CFH expression was significantly decreased in the hippocampi of TLE patients. It is unfortunate that we used cells replacing the *CFH* gene knockout TLE rats owing to available funds and time. We plan to use the *CFH* gene knockout TLE rats to test the results again in the future. After injection with miR-146a or IL-1β, rats were monitored by EEG for only 2 days as they were sacrificed 48 h later. The EEG changes were obvious, but it was a drawback that there was no increase in convulsive motor seizures after miR-146a or IL-1β injection, possibly as a result of the small quantity of miR-146a or IL-1β and the short duration of monitoring. It is evident that most studies indicate that miR-146a is a key regulator of immune and inflammatory signaling and can be induced by pro-inflammatory cytokines (Taganov et al., 2006; Lukiw et al., 2008; Nakasa et al., 2008; Iyer et al., 2012). Furthermore, miR-146a and pro-inflammatory cytokine IL-1β are jointly increased in the chronic phase in TLE rats and TLE patients (Aronica et al., 2010; Omran et al., 2012), which suggests that miR-146a can promote inflammation in TLE. These findings are consistent with our study results. However, a recent study that delivered miR-146a mimic intranasally before pilocarpine injection to C57BL/6 mice found that it can improve seizure onset and hippocampal damage in the acute phase and even support an anti-inflammatory role for miR-146a (Tao et al., 2017). The findings were inconsistent with those from most studies, including our study, which might be caused by differences in the drug administration pathway and model establishment method. However, the exact mechanism is worth further investigation.

Overall, this study provides evidence for how miR-146a modulates the inflammatory signaling occurring in TLE, and we detected, for the first time, that CFH expression was significantly decreased in the hippocampi of TLE patients. The results show that enhancive miR-146a can upregulate IL-1β levels in chronic TLE by downregulating CFH, and that upregulation of IL-1β plays an important feedback-regulating role in the expression of miR-146a and CFH. Consequently, the miR-146a–CFH–IL-1β inflammatory loop circuit seems to provoke an amplification cascade of inflammation, and the upregulation of the upper or lower reaches of miR-146a/CFH/IL-1β levels is associated with further increases in inflammatory factors and hyperexcitability. This loop circuit might lead to the perpetuate inflammation in TLE, which could explain the drug resistance in TLE; however, this mechanism remains to be investigated further. Therefore, modulation of the miR-146a–CFH–IL-1β loop circuit might be a novel therapeutic target for TLE. Moreover, the potential clinical application of CFH research in TLE is using biofluid profiles as molecular biomarkers, which will allow monitoring of the dynamics of TLE and improvement of the diagnostic/prognostic accuracy, as has been performed in AD (Song et al., 2009).

## MATERIALS AND METHODS

### Animals

Specific pathogen-free (SPF) adult male Sprague-Dawley (SD) rats (250-280 g) were obtained from Beijing Vital River Laboratory Animal Technology Company Limited (Beijing, China) and kept under SPF conditions. All animal studies were performed according to the guidelines of the Guidance for Animal Experimentation of Capital Medical University, and the protocol was approved by the Experimental Animal Ethics Committee of Beijing Tiantan Hospital, Capital Medical University (Permit No. 201502002). All surgeries were performed under chloral hydrate anesthesia.

### Seizure induction and electrode implantation

SPF adult male SD rats (250-280 g) were anesthetized with 10% chloral hydrate (0.3 ml/kg) intraperitoneally and then mounted in a stereotaxic frame (David Kopf Instruments, USA). Then, 0.8 µg (1 µg/µl in saline) KA was injected in the CA3 region of left hippocampus with the following coordinates [on the basis of the rat brain atlas of Paxinos and Watson (2007)]: anterior posterior (AP), −5.64 mm; mediolateral (ML), 4.7 mm; dorsoventral (DV), 6.8 mm, relative to bregma. The injection was conducted with a 1.0-µl microsyringe (RWD Life Science & Technology, China) and persisted for 5-10 min to avoid leaks of the solution. To record the hippocampal EEG, 25% of the rats also had electrodes and catheters implanted. When the skull was exposed, three extra holes were drilled (4.0 mm posterior and 3.0 mm left and right of the bregma; center of the occipital), and electrode leads were attached to the skull bone with stainless steel screws (62510, RWD Life Science & Technology) in an epidural position. A catheter and cap (62001 and 62101, RWD Life Science & Technology) were inserted into the KA injection hole vertically. The depth of the catheter implanted was 6.8 mm to ensure that it reached the CA3 region. Finally, the surface of the surgical site (i.e. skull, screws, electrode leads and catheters) was covered and sealed with dental cement.

### Video and EEG monitoring of seizures

After surgery, all rats were continuously monitored for behavioral seizures in the first 24 h with video recording in the cages. Additionally, the electrode-implanted rats also received EEG monitoring in the first 24 h. The severity of behavioral seizures was scored according to the modified Racine scale (Racine, 1972) as follows: I, mouth and facial movements; II, head nodding; III, forelimb clonus and a lordotic posture; IV, rearing with forelimb clonus; and V, rearing, forelimb clonus and falling. SE was defined as the onset of continuous generalized (score 4-5) seizure activity lasting no less than 40 min, and only those animals that reached SE were included in this study. The KA-induced SE was not terminated pharmacologically, and no special care was given to the individuals.

The rats that survived from the acute phase were raised in cages with adequate water and food. Starting from week 5 (chronic phase) post-SE, the rats received video monitoring 13 h/day (19:00-08:00) and were observed in the afternoon (13:00-19:00). The video recording lasted until the end of week 6. The electrode-implanted rats also received EEG monitoring in the afternoon. We also used the modified Racine scale (Racine, 1972) to observe spontaneous recurrent seizures (SRS), which is widely accepted (Williams et al., 2009; Rattka et al., 2013). Sudden immobility, Racine Class I and Class II seizures were typically described as nonconvulsive seizures. The corresponding type of seizure appeared on EEG as a rhythmic train of spike-waves, which began abruptly and lasted for tens of seconds (Campbell et al., 2014), so characteristic electrographic seizures were used to identify them such that they could be a model for complex partial seizures. Racine Class III, IV and V seizures are characterized as convulsive motor seizures. All convulsive motor seizures were associated with EEG seizures and could be identified by observation; they would be considered generally similar to a clinical seizure in a human patient. Both electrographic nonconvulsive and convulsive seizures were considered as SRS (Williams et al., 2009). Approximately 71% of the rats had SRS. The rats were eliminated if they had no SRS or their electrodes abscised.

Rats with seizures were randomly assigned to five groups at week 7: (1) the IL-1β control group (*n*=10, rats were injected with 1 μl 0.9% saline); (2) the IL-1β group (*n*=10, rats were injected with 1 μl 50 ng/μl IL-1β, three electrode-implanted rats); (3) the miR-146a group (*n*=10, rats were injected with 1 nmol miR-146a agomir, three electrode-implanted rats); (4) the miR-146a sponge group (*n*=10, rats were injected with 1 nmol miR-146a antagomir); and (5) the miR-146a control group (*n*=10, rats were injected with 1 nmol miR-146a control). After injection, the rats were continuously video monitored for 2 days, and the electrode-implanted rats were monitored with EEG at the same time before injection. Forty-eight hours after injection, the rats were sacrificed and hippocampal tissues collected.

EEG was recorded using a multi-channel amplifier (UEA-B, Symtop, China) that was bandpass filtered between 0.1 and 120 Hz, digitized with 1000 samples/s/channel and stored on a hard disk. In the chronic phase of the epileptic model, 30-min samples of electrical activity were selected for further analysis at each of the following timepoints: 2 days before injection of IL-1β or miR-146a agomir, 1 day before injection of IL-1β or miR-146a agomir, 1 day after injection of IL-1β or miR-146a agomir, and 2 days after injection of IL-1β or miR-146a agomir. All samples were taken during periods of waking, with small movement disturbance. The analysis was performed by time-frequency analysis to extract frequency bands spanning 1-60 Hz, the frequency range currently used in clinical practice, and we calculated average spectral power using EDF browser software (obtained from https://www.teuniz.net/edfbrowser/).

### Tissue collection

In each group, all rats were decapitated for WB and PCR analysis. The brains were rapidly dissected, and the left hippocampus was removed and divided equally into two parts along the long axis of the hippocampus to ensure that each part contained all regions. Tissues were rapidly cooled in liquid nitrogen for more than 30 min and stored at –80°C until use. Each group of animals was processed independently, and the whole process was carried out in ice saline to avoid RNA degradation and cross-contamination.

### Cell cultures and transfections

U251 (human astrocytoma) cells (obtained from the National Infrastructure of Cell Line Resource, Beijing, China) were grown in Dulbecco’s modified Eagle medium (DMEM, Thermo Fisher Scientific, USA) supplemented with 10% fetal bovine serum (FBS; Gibco, Thermo Fisher Scientific) and 2 mmol/l L-glutamine (Amresco, USA). They were authenticated before experiments and not contaminated after testing.

Then, 2×10^5^ U251 cells were seeded into 24-well plates. After 12 h, U251 cells were transfected with three *CFH*-specific siRNA or siRNA control (single gene suit of siRNA, RiboBio, China). Twenty-four hours post-transfection with *CFH*-specific siRNA, U251 cells were transfected with miR-146a mimic. Thirty-six hours post-transfection with the miR-146a mimic, the cells were harvested for RNA isolation. The transfection time was confirmed by preliminary experiments and the protocol.

### TLE patients and controls

This study was approved by the Institutional Ethics Committee of Beijing Tiantan Hospital, conforming with the principles expressed in the Declaration of Helsinki. Written informed consent was obtained from all patients and control participants. Tissue samples were obtained at surgery from seven patients with unilateral drug refractory TLE, and typical imaging features and pathological confirmation of HS (four left and three right), who had unilateral selective amygdalohippocampectomy (Table S1). Surgical samples were subjected to routine histopathological examination. Eight normal hippocampal samples as controls were obtained from patients with no history of epilepsy (gifted from Peking Union Medical College Hospital) (Table S2). All autopsies were performed within 8 h after death. Neuropathologic examination confirmed that the control tissues were normal. Clinical information on patients with TLE and controls is shown in Tables S1 and S2.

### RNA isolation and qRT-PCR

The total RNA of the hippocampal or U251 cells was isolated according to the manufacturer's protocol (TRIzol reagent, Invitrogen, USA). For CFH, IL-1β and β-actin, 2.5 μg (tissue) or 1.0 μg (cell) of total RNA were reverse-transcribed into cDNA using oligo (dT) primers according to the GoScript Reverse Transcription System (A5001, Promega, USA). For miR-146 and U6, 4.0 μg (tissue) or 2.0 μg (cell) of total RNA were reverse-transcribed into cDNA by polyA polymerase according to the manufacturer's instructions (All-in-one™ miRNA qRT-PCR Detection Kit, GeneCopoeia, USA).

Oligonucleotides used for qRT-PCR were synthesized for amplifying cDNA in SD rats and human samples are shown in Table S3. *β-actin* or *U6* was used as the housekeeping gene. For each PCR, the master mix was prepared on ice containing 1 μl cDNA (diluted five times with RNase-free water), 10 μl of 2×SuperReal PreMix Plus (FP205, TianGen Biotech, China), and 0.3 μM of both forward and reverse primers in each sample. The final volume was adjusted to 20 μl with RNase-free water. For miR-146a and U6, each sample contained 2 μl cDNA (diluted five times with RNase-free water), 10 μl mix (All-in-one™ miRNA qRT-PCR Detection Kit, GeneCopoeia), and 0.2 μM of both forward and reverse primers. The final volume was adjusted to 20 μl with RNase-free water. We evaluated every sample in triplicate to correct operating errors. The reaction conditions were as follows: initial denaturation at 95°C for 10 min, followed by 45 cycles of denaturation at 95°C for 10 s, annealing at 58°C for 20 s and extension at 72°C for 10 s. The fluorescent product was measured by a single acquisition mode at 72°C after each cycle. A melting curve was obtained as mentioned above. Data were quantified using a modification of the 2^-ΔΔCT^ method as described previously (Livak and Schmittgen, 2001).

### WB and quantification

Hippocampi stored at –80°C were weighed and homogenized with a glass tissue grinder, and U251 cells were lysed with cell lysis buffer. The supernatant was centrifuged (12,000 ***g*** for 5 min). Protein content was detected by a BCA protein assay kit (Enhanced BCA Protein Assay Kit, Beyotime, China). Protein separation was performed under reducing conditions (with 100 mmol/l 2-mercaptoethanol and boiling) in 10% sodium dodecyl sulfate poly-acrylamide gel electrophoresis (SDS-PAGE) gel. After blotting onto nitrocellulose membranes (Whatman, UK), proteins were incubated at 4°C overnight with specific primary antibodies: anti-CFH (1:500, ab8842, Abcam, UK), anti-IL-1β (1:500, 16806-1-AP, Proteintech, USA) and anti-β-actin (1:1000, TA-09, Zsgb-Bio, China). After washing, the membrane was incubated with horseradish peroxidase-coupled secondary antibody (Biodragon Immunotechnologies, China) and visualized by enhanced chemiluminescence (Invitrogen, USA). WB quantitation was performed using ImageJ software (NIH). Details on antibody validation are provided in the Supplementary Information.

### Statistical analysis

Statistical analyses were performed with SPSS 13.0 software (SPSS, Chicago, IL, USA). All data are expressed as means±s.d. Differences were evaluated by ANOVA. *P*<0.05 was considered statistically significant.

## Supplementary Material

Supplementary information
