# Supplementary material for: The role of the microRNA-146a/complement factor H/interleukin-1β-mediated inflammatory loop circuit in the perpetuate inflammation of chronic temporal lobe epilepsy
Source: Dis Model Mech. 2018 Mar 1;11(3):dmm031708. doi: 10.1242/dmm.031708 (PMC5897725; doi:10.1242/dmm.031708)
Supplement: Supplementary information [file dmm-11-031708-s1.pdf]

Supplemental File

Table S1. Clinical information on the patients with TLE

ID	Sex	Age (years)	Duration of epilepsy (years)	Side of hippocampus sample	Antiepileptic drug (AED)
Patient 1	M	43	28	Right	PB+CBZ+OXC
Patient 2	F	24	8	Left	CBZ+TPM+LEV
Patient 3	F	29	16	Left	PB+CBZ+PHT+CZP
Patient 4	M	19	7	Left	TPM+VPA+LTG
Patient 5	F	32	23	Right	LTG+CZP+TPM
Patient 6	F	28	14	Left	CBZ+TPM
Patient 7	F	20	7	Right	VPA+CZP+LTG

Abbreviations: TLE, temporal lobe epilepsy; TPM, topiramate; CZP, clonazepam; VPA, valproate; PB, phenobarbitone; CBZ, carbamazepine; OXC, oxcarbazepine; LTG, lamotrigine; PHT, phenytoin; LEV, levetiracetam.

Table S2. Clinical information on controls

ID	Sex	Age (years)	Side of hippocampus sample	Causes of death
Control 1	M	26	Left	Schizophrenia (commit suicide by jumping off a high building)
Control 2	F	44	Left	Mammary cancer
Control 3	F	16	Left	Leukaemia
Control 4	F	46	Left	Cervical carcinoma
Control 5	M	42	Right	Rectal cancer
Control 6	M	31	Right	Cerebral infarction
Control 7	F	22	Right	Heart disease
Control 8	F	48	Right	Choriocarcinoma

Table S3. Oligonucleotides used for qRT-PCR

Gene	Type	Sequence (5’ to 3’)
SD rats CFH	Forward	AATTTCTTGCCTGCCACCAC
	Reverse	GCAGACAGCATCCCCTCTTT
SD rats IL-1β	forward	AGGCTTCCTTGTGCAAGTGT
	reverse	TGAGTGACACTGCCTTCCTG
SD rats β-actin	forward	GCCAACACAGTGCTGTCTG
	reverse	TACTCCTGCTTGCTGATCCA

SD rats miR-146a	forward	TGAGAACTGAATTCCATGGGTT
	reverse	The reverse was the universal adaptor PCR primer in the kit
SD rats U6	forward	CGCAAATTCGTGAAGCGTTC
	reverse	The reverse was the universal adaptor PCR primer in the kit
Human CFH	forward	TCCAGAAGGCACCCAGGCTATCTA
	reverse	CCACAGGGCCTTTTCTGACATTTCC
Human IL-1β	forward	AGTGGCAATGAGGATGACTTGT
	reverse	AGATGAAGGGAAAGAAGGTGCT
Human β-actin	forward	CTGGGACGACATGGAGAAAA
	reverse	AAGGAAGGCTGGAAGAGTGC
Human miR-146a	forward	Synthesized by GeneCopoeia™
	reverse	The reverse was the universal adaptor PCR primer in the kit
Human U6	forward	Synthesized by GeneCopoeia™
	reverse	The reverse was the universal adaptor PCR primer in the kit

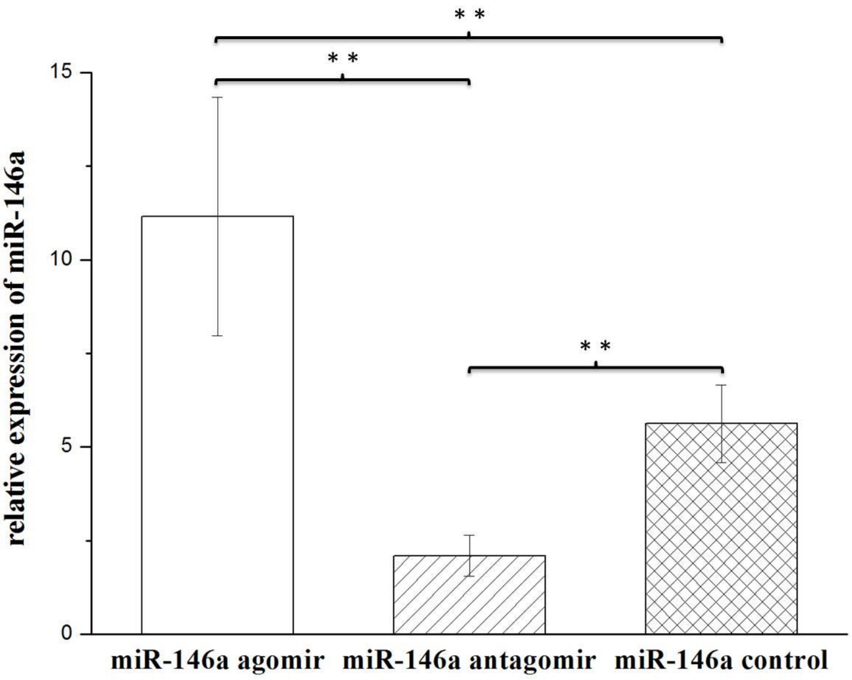

Fig. S1 Expression of miR-146a in the hippocampi of chronic TLE rats after injection with miR-146a agomir (n=10), miR-146a antagomir (n=10) or control (n=10). The analysis was conducted by one-way ANOVA, followed by LSD test. $^{**}P < 0.01$. Values represent means ±SD. At the fifth and sixth week (chronic phase) post-SE, all rats received video monitoring, and electrode-implanted rats also received EEG monitoring. All rats experienced seizures, and abnormal seizure waves were found in electrode-implanted rats. Rats were injected with miR-146a

Disease Models & Mechanisms • Supplementary information

agomir, miR-146a antagomir or control at the seventh week (chronic phase) post-SE and executed to collect hippocampal tissues 48 hours later. Expression of miR-146a was detected by qRT-PCR. The expression was normalized to *U6* in each tissue. Compared with miR-146a antagomir or control group, miR-146a expression was up-regulated in the miR-146a agomir group. Compared with control group, miR-146a expression was down-regulated in the miR-146a antagomir group.

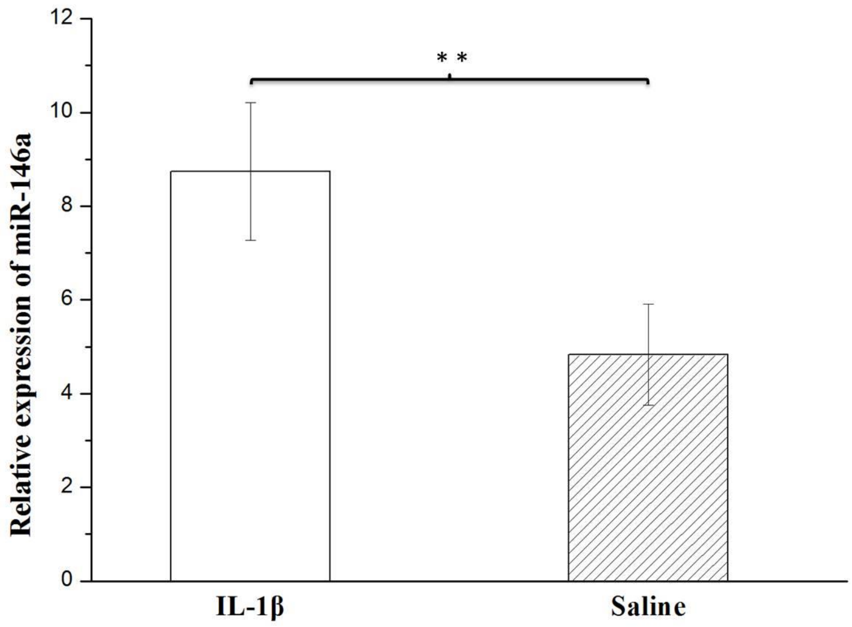

Fig. S2 Expression of miR-146a in the hippocampi of chronic TLE rats after injection with saline (n=10) or IL-1β (n=10). The analysis was conducted by one-way ANOVA. **P < 0.01. Values represent means ±SD. At the fifth and sixth week (chronic phase) post-SE, all rats received video monitoring, and electrode-implanted rats also received EEG monitoring. All rats experienced seizures, and abnormal seizure waves were found in electrode-implanted rats. Rats were injected with IL-1β or saline at the seventh week (chronic phase) post-SE and executed to collect hippocampal tissues 48 hours later. Expression of miR-146a was detected by qRT-PCR. The expression was normalized to *U6* in each tissue. Compared with the control group, miR-146a expression in the IL-1β group was up-regulated.

The anti-CFH (ab8842, Abcam, UK) was validated in many articles (PubMed: 26654980, 26200783, 25299043, etc).
The anti-IL-1β (16806-1-AP, Proteintech, US) was validated in many articles (PubMed: 23603407, 23809162, 24687774, etc).

Disease Models & Mechanisms • Supplementary information
